# *Daphne Genkwa* Sieb. et Zucc. Water-Soluble Extracts Act on Enterovirus 71 by Inhibiting Viral Entry

**DOI:** 10.3390/v4040539

**Published:** 2012-04-11

**Authors:** Chia-Wen Chang, Yan-Lii Leu, Jim-Tong Horng

**Affiliations:** 1 Department of Biochemistry, Chang Gung University, Kweishan, Taoyuan 333, Taiwan; Email: anny910111@hotmail.com; 2 Graduate Institute of Natural Products, Chang Gung University, Kweishan, Taoyuan 333, Taiwan; Email: ylleu@mail.cgu.edu.tw; 3 Chinese Herbal Medicine Research Team, Healthy Aging Research Center, Chang Gung University, Kweishan, Taoyuan 333, Taiwan; 4 Research Center for Emerging Viral Infections, Chang Gung University, Kweishan, Taoyuan 333, Taiwan

**Keywords:** attachment assay, cytopathic effect, enterovirus 71, *Daphne Genkwa* Sieb. et Zucc., penetration assay, yuanhua

## Abstract

Dried flowers of *Daphne genkwa* Sieb. et Zucc. (Thymelaeaceae) are a Chinese herbal medicine used as an abortifacient with purgative, diuretic and anti-inflammatory activities. However, the activity of this medicine against enteroviral infections has not been investigated. The water-extract of dried buds of *D. genkwa* Sieb. et Zucc. (DGFW) was examined against various strains of enterovirus 71 (EV71) by neutralization assay, and its initial mode of action was characterized by time-of-addition assay followed by attachment and penetration assays. Pretreatment of DGFW with virus abolished viral replication, indicating that DGFW inhibits EV71 by targeting the virus. GFW exerts its anti-EV71 effects by inhibiting viral entry without producing cytotoxic side effects and thus provides a potential agent for antiviral chemotherapeutics.

## 1. Introduction

Enterovirus 71 (EV71) is a positive-strand RNA virus and belongs to the *Enterovirus* genus of the family *Picornaviridae*. It is a member of the Picornaviridae family and has a (+)RNA genome 7.5 kb in length that encodes 2193 amino acids [1,2]. The polyprotein is cleaved proteolytically into distinct proteins by the viral-encoded proteases 2A^pro^ and 3C^pro^. The structural proteins comprising VP1 to VP4 and the nonstructural proteins 2A to 2C and 3A to 3D are required for viral replication [2]. EV71 is a common enterovirus that usually causes hand, foot and mouth disease in children but is often associated with brainstem encephalitis, neurogenic shock and polio-like acute flaccid paralysis. EV71 caused an outbreak with 78 mortalities in 1998, with manifestations of neurological involvement and cardiopulmonary failure. In a long-term study, survivors of EV71-induced CNS infection were found to have neurological sequelae and retarded mental development [3]. Several epidemics occurred and caused a total of 51 fatalities in 2000–2001 [4]. Unfortunately, there is no therapy with proven efficacy to control EV71. Given these recurring outbreaks and annual epidemics, there is a clear need to develop inhibitors for EV71.

Viruses must deliver their genome into the host cells to initiate replication. Most of them enter cells by endocytosis during which they hijack cellular machinery for the attachment to receptors and subsequent penetration across the plasma membrane. Several receptors to facilitate EV71 entry have been reported, including scavenger receptor B2, human P-selectin glycoprotein ligand-1 and sialylated glycans [5,6,7]. A strategy to disrupt the interaction between viral particles and their receptors would be ideal for antiviral development.

We have been identifying traditional Chinese medicines with antiviral or prophylactic activity against EV71 infections [8,9]. The flower buds of *Daphne genkwa* Sieb. et Zucc. (Thymelaeaceae), known as Won-Hwa or Yuanhua in Chinese, are collected in the spring before blossoming. As a traditional medicine, they have been used as an abortifacient [10], with purgative, diuretic and anti-inflammatory actions [11]. Previous phytochemical studies on *D. genkwa* have led to the isolation of pharmacologically active flavonoids, diterpene orthoesters and coumarins, showing inhibitory activity against xanthine oxidase and adenosine 3´,5´-cyclic monophosphate phosphodiesterase, as well as exhibiting antileukemic activity [12]. In the present study, we used Chinese traditional medicine extracts for anti-EV71 activity. We found that *Daphne genkwa* Sieb. et Zucc. extracts (DGFW) could neutralize the EV71-induced cytopathic effect (CPE) in human embryonic rhabdomyosarcoma (RD) cells. The half maximal effective concentration (EC_50_) of DGFW on EV71 was approximately 0.163 ± 0.013 mg/mL. DGFW inhibited EV71 viral protein and viral RNA synthesis. Additionally, DGFW suppressed viral activity at the adsorption stage of uptake as shown by a time-of-addition assay. Cells pretreated with DGFW could not block viral infection. Therefore, it is possible that DGFW binds to the virus to affect its activity. We conclude that DGFW possesses antiviral activity and has the potential for development as an anti-EV71 agent.

## 2. Results and Discussion

### 2.1. The Water-Soluble Fraction of D. genkwa Inhibits Viral Replication

**Figure 1:**
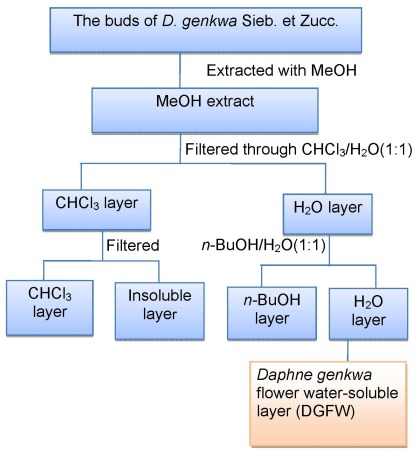
Flow chart of preparation of water-soluble fractions of *D. genkwa*.

In an endeavor to identify anti-EV71 Chinese medicines, we initially screened a panel of 48 extracts of Chinese herbs with antioxidant properties. An assay to measure the inhibition of virus-induced cell death in RD cells was first employed using 1 mg/mL of herbal extracts followed by EC_50_ determination if the candidates possessed antiviral activity from the primary screening. We identified that the water fraction of *D. genkwa* was a potent agent against EV71 isolate 2231 with an EC_50_ of 0.163 ± 0.013 mg/mL and a concentration producing 50% cytotoxicity (CC_50_) of 1.444 ± 0.193 mg/mL using RD cells (Table 1). However, its solvent (CHCl_3_) layer did not demonstrate anti-EV71 activity (Figure 1). DGFW was also able to inhibit successfully other isolates of EV71 except for isolate 1101 (genotype B5; Table 1). Interestingly, this extract was effective against influenza virus, although the selectivity index was not satisfactory. However, DGFW did not show activity against CVs or two DNA viruses, adenovirus and HSV-1, at a concentration of 1 mg/mL (Table 1).

**Table 1:** Viral inhibition spectrum of *D. genkwa* Sieb. et Zucc. (DGFW).

Title	Concentration (mg/mL)		
	CC_50_^a^	EC_50_^b^	SI ^c^
Cytotoxic effect			
RD cells	1.444 ± 0.193		
MDCK cells	1.603 ± 0.091		
HFF3 cells	2.354 ± 0.018		
A549 cells	>2		
EV71^d^, TW/2231/98 (genotype C)		0.163 ± 0.013	8.859
EV71, BrCr (genotype A)		0.182 ± 0.027	7.934
EV71, TW/71552/05 (subgenotype C4)		0.824 ± 0.004	1.752
EV71, TW/1101/08 (subgenotype B5)		>1	–
CV-B1 ^d^		>1	–
CV-B2		>1	–
CV-B3		>1	–
CV-B4		>1	–
CV-B5		>1	–
Influenza virus A/WSN/33		0.838 ± 0.026	1.913
Adenovirus		>2	–
HSV-1 ^d^		>2	–

^a^ CC_50_ was determined by MTT assay; ^b^ EC_50_ was determined by inhibiting the virus-induced CPE. Different isolates of enterovirus (EV)71 and coxsackieviruses (CV) were assayed in RD cells; adenovirus and HSV-1 were assayed in A549 cells; and influenza virus was assayed in MDCK cells; ^c^ SI (selectivity index) is the ratio of CC_50_ to EC_50_; ^d^ EV71: enterovirus 71; CV: Coxsackievirus; HSV, herpes simplex virus.

**Figure 2:**
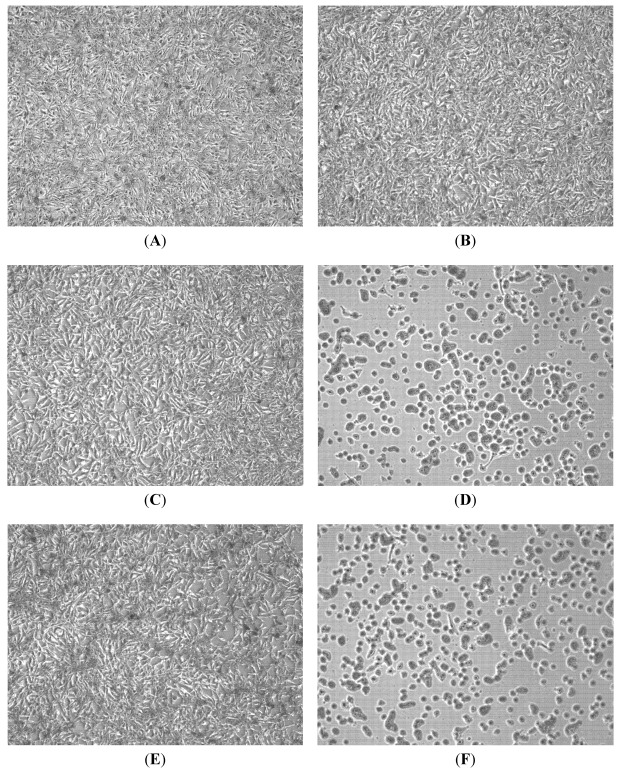
Inhibition of enterovirus 71 (EV71)-induced cytopathic effect (CPE) by DGFW. rhabdomyosarcoma (RD) cells were preadsorbed with EV71 (m.o.i. 1) in the presence or absence of 0.5 mg/mL DGFW for 1 h at 4 °C. The culture medium was removed and replaced with DMEM containing 2% FBS with or without DGFW. After 20 h, the cells were fixed and stained with SRB, followed by microscopy.(**A**) Mock; (**B**) Mock + 0.5 mg/mL; (**C**) Mock + 0.25% DMSO; (**D**) Virus; (**E**) Virus + 0.5 mg/mL DGFW; (**F**) Virus + 0.25% DMSO.

**Figure 3:**
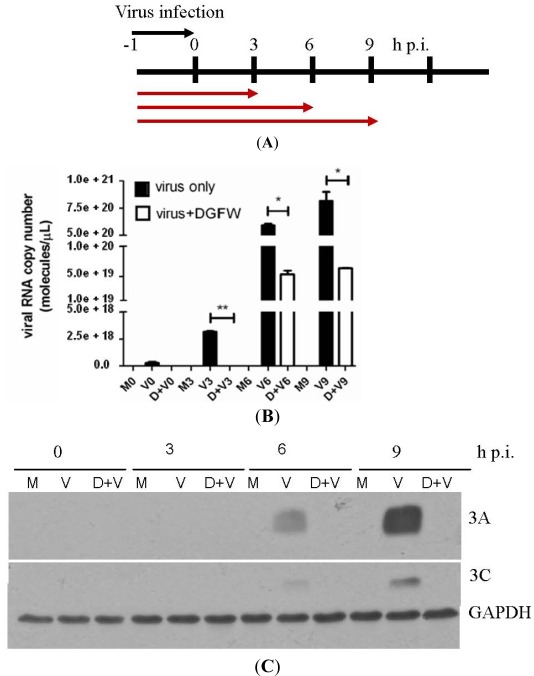
DGFW inhibits viral protein and RNA synthesis. (**A**) An illustration of the treatment with DGFW. Cells were preadsorbed with EV71 at an m.o.i. of 5 in the presence of 0.5 mg/mL DGFW. Culture supernatants were then replaced with DMEM containing 2% FBS and DGFW. The cells were preadsorbed with virus at 4 °C between −1 to 0 h p.i. The cells were then harvested at 0, 3, 6 and 9 h p.i. for qPCR (**B**) and western blot (**C**) analysis. The quantity of viral RNA was quantified against a standard curve of a serial dilution of viral RNA. M and D indicate mock-infected cells and drug-treated cells, respectively. These are representative data from three independent experiments. The data were analyzed using two-tailed Student’s *t*-tests and are expressed as the mean ± SEM. * *p* < 0.05, ** *p* < 0.01 and *** *p* < 0.001.

The preparation of DGFW is shown in Figure 1 and the final dried extract was weighed and dissolved in DMSO for antiviral analysis. We explored the effects of DGFW in protecting against virus-induced CPE on RD cells by microscopic assay (Figure 2). The RD cells were infected with EV71 isolate 2231 in the presence or absence of 0.5 mg/mL, which was not toxic to the cells by MTT assay (Figure S1). RD cells infected with virus displayed a typical CPE in which the cells became rounded and eventually detached from the culture dish (panel D, Figure 2). This effect was not caused by DGFW alone (panel B, Figure 2) or 0.25% vehicle DMSO (panel C, Figure 2). DGFW but not DMSO protected the cells from virus-induced CPE (panel E *versus* F, Figure 2), indicating that DGFW inhibited viral replication. We therefore performed qPCR and western blotting to confirm the antiviral effect of DGFW (Figure 3). The cells were first infected with virus in the presence or absence of DGFW and the cells were harvested at indicated points (Figure 3A). The amount of viral RNA was quantified against a standard curve of known concentrations. The synthesis of viral protein was measured as viral proteins 3A and 3C. DGFW substantially reduced the synthesis of viral RNA and viral proteins (Figure 3B,C).

### 2.2. DGFW Inhibits Early Stages of Replication

**Figure 4:**
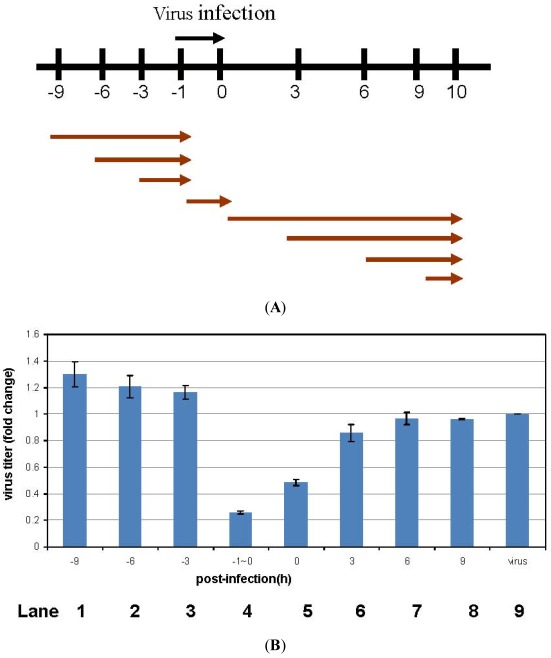
Time-of-addition assay of DGFW. (**a**) RD cells were infected with EV71 (m.o.i. 5). DGFW(0.5 mg/mL) was added at the designated times. (**b**) Viral adsorption was from −1 to 0 h p.i. on ice. DGFW (0.5 mg/mL in E2) was added to the cells at the indicated times. After each treatment, DGFW was washed and replaced with fresh E2. RD cells were pretreated with DGFW from −9 to −1, −6 to −1, or −3 to −1 h p.i. The cells were washed and inoculated with EV71 for 1 h, and the unbound virus was removed and replaced with E2 and incubated for an additional 10 h. The supernatants were collected and the viral yields determined by plaque assay; “virus” is a virus-only control without DGFW treatment. The ratio of viral titers is presented as the mean ± SEM of the results of two experiments, and this is normalized to the virus-only control, set to 1. This is one representative result from two independent experiments.

We assessed the antiviral mode of action using a time-of-addition assay in which DGFW was added at different stages of one infection cycle (Figure 4). DGFW was either added before viral preadsorption, during adsorption (–1 to 0 h p.i.), or after viral adsorption. The viruses from cells and culture medium were then collected for plaque assay at 10 h p.i. (Figure 4B). DGFW was able to reduce viral titer considerably during the viral preadsorption step between −1 and 0 h p.i. (lane 4, Figure 4). DGFW did not target the cells when it was added before viral preadsorption (lanes 1–3, Figure 4B). The late stages, such as viral package and release, were not inhibited by DGFW because no reduction of viral titer was seen when the extract was added immediately after viral adsorption (lanes 6–8, Figure 4B). Altogether, these results demonstrate that DGFW might target cellular uptake of the virus.

### 2.3. DGFW Inhibits Viral Attachment and Penetration

**Figure 5:**
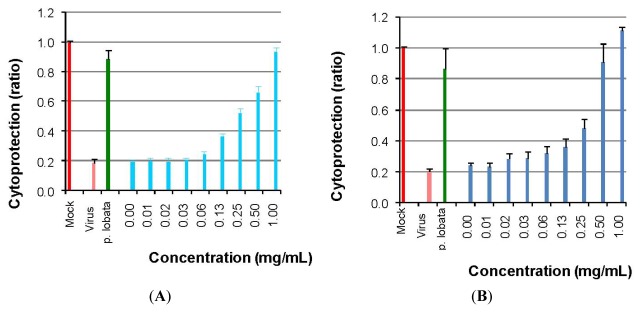
Inhibition of viral entry by DGFW. Human foreskin fibroblast (HFF3) cells were cultured in 96-well plates. Cytoprotection was then measured by crystal violet staining of the attached cells on the plates. A *Pueraria lobata* extract at 3.125 mg/mL served as a positive control. (**A**) Dose dependence of DGFW inhibition of EV71 attachment. Increasing concentrations of DGFW were added to cells infected with EV71 and incubated for 3 h on ice. The cells were washed with DPBS and replaced with E2, warmed to 37 °C and incubated for 72 h. Cytoprotection was then determined. (**B**) Dose dependence of DGFW inhibition of EV71 penetration. HFF3 cells were chilled on ice for 1 h, followed by preadsorption of EV71 for 3 h on ice. Increasing concentrations of DGFW were then added, warmed to 37 °C and incubated for 1 h to allow the virus to enter the cells. The monolayers were treated with alkaline PBS (pH 11) to inactivate extracellular virus and were incubated at 37 °C for 72 h. Cytoprotection was then determined. This is a representative result from three independent experiments.

To test the mechanism of inhibition of viral entry into host cells by DGFW, we performed attachment and penetration assays. Viral uptake into cells occurs first by binding to receptors followed by clathrin-dependent endocytosis into early endosomes [13]. The attachment assay was used to examine the binding of the virus to receptors, and the penetration assay was used to test the entry step following receptor binding. We used HFF3 cells for these assays because RD cells were easily detached from the dishes when incubated on ice, and HFF3 cells are susceptible to EV71 infection [14]. For the attachment assay, the HFF3 cells were incubated with 100 TCID_50_ of EV71 for 3 h in the presence of DGFW on ice. The cytoprotection of DGFW was then determined. DGFW was able to inhibit attachment of EV71 into HFF3 cells with an EC_50_ of 0.185 ± 0.056 mg/mL (Figure 5A). For the penetration assay, DGFW was added during viral entry into cells at 37 °C following viral preadsorption. DGFW was also able to inhibit penetration with an IC_50_ of 0.245 ± 0.063 mg/mL (Figure 5B). DGFW might bind to and thus inhibit the virus, because DGFW displayed a similar IC_50_ against EV71 based on attachment and penetration assays.

### 2.4. DGFW Targets the Virus Directly but not the Host Cells

**Figure 6:**
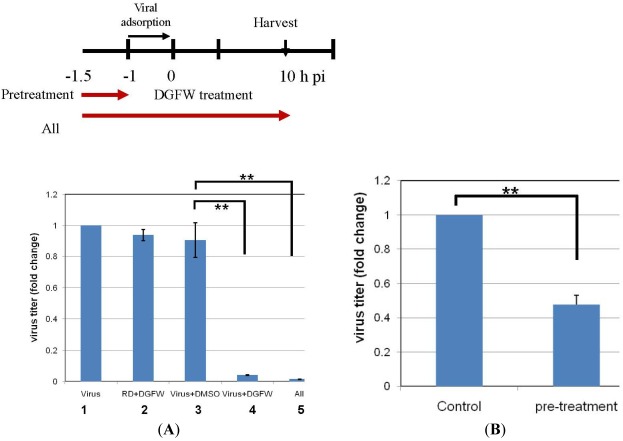
DGFW might target the virus directly. (**A**) EV71 (m.o.i. 5) was preincubated with DGFW (0.5 mg/mL) or control vehicle (DMSO) for 30 min at 37 °C (−1.5 to −1 h. p.i.) and was then used to inoculate RD cells for 1 h (adsorption at −1 to 0 h p.i.). Similarly, RD cells were pretreated with DGFW for 30 min before viral adsorption for 1 h (–1 to 0 h p.i.). At 10 h p.i., the cells and culture medium were pooled for the determination of viral titers by plaque assays. “All” indicates the presence of DGFW all through from −1.5 to 10 h p.i.; “virus” is a nontreated virus control, and the titer was set to 1. The fold change of viral titers is presented as the mean ± SEM of the results of two independent experiments. ** *p* < 0.01; (**B**) EV71 was preincubated with DGFW (1 mg/mL) or DMSO for 30 min at 37 °C. The mixture was diluted 10^5^-fold for the plaque assay. The viral titer from pretreatment with DMSO was set to 1, and the fold change of viral titers is presented as the mean ± SEM of the results of two independent experiments. ** *p* < 0.01.

We next examined whether DGFW acted at the cell surface, such as via receptor–virus binding, by pretreatment of RD cells with DGFW for 30 min at 37 °C (lane 2, Figure 6). Unbound DGFW was removed, the cells were then infected with EV71 for 1 h, and the progeny viruses in the cells and culture medium were harvested for plaque assay at 10 h p.i. Similarly, EV71 was pretreated with DGFW or DMSO for 30 min at 37 °C, and the infectivity of the treated virus was then determined by plaque assay (Figure 6). Pretreatment of cells with DGFW did not show any reduction of viral titer, suggesting that DGFW did not affect viral binding to the cells directly. In contrast, pretreatment of the virus with DGFW caused a substantial reduction of viral titer, but this was not seen after treatment with DMSO alone, implying that DGFW might target the virus directly and specifically (Figure 6; lane 3 *versus* lane 4). This result was consistent with the time-of-addition assay (Figure 4). This direct targeting effect was further demonstrated by a lower concentration of DGFW. This was done by pretreating the virus with DGFW for 30 min followed by diluting the mixture 10^5^-fold, whereby the DGFW did not have inhibitory activity. The infectivity of the treated virus was then determined by plaque assay (Figure 6B). Pretreatment with DGFW produced a significant reduction of viral titer by about 50% compared with the DMSO control, suggesting again that DGFW suppresses viral activity by targeting the virus directly (Figure 6B).

### 2.5. Discussion

In this study, we demonstrated that DGFW suppressed viral adsorption to RD cells by time-of-addition assay. Cells pretreated with DGFW did not show inhibition of viral infection, indicating that DGFW does not inhibit cellular viral receptors. However, DGFW was able to inhibit viral endocytosis by reducing viral attachment to and penetration into host cells. Furthermore, pretreatment of virus with DGFW abolished viral replication, indicating that DGFW inhibits EV71 by targeting the virus directly (Figure 6).

Currently, there is no effective vaccine or antiviral agent to combat EV71. Many antienteroviral agents against poliovirus or rhinovirus have been thus tested for their efficacy against EV71. Pleconaril, a WIN compound and in clinical development, has been reported to inhibit several types of enterovirus-induced meningitis and aseptic meningitis [15]. The WIN series of compounds inhibit viral replication primarily by binding the hydrophobic canyon region of capsid protein VP1 to stabilize the viral capsid structure, so that after the virus enters the host cell it cannot undergo the uncoating process [16]. However, Pleconaril did not effectively inhibit the CPE induced in cell cultures by the EV71 strain isolated from patients who had contracted hand, foot and mouth disease in 1998 [17]. We demonstrated here that DGFW might target the viral particle for inhibition. However, DGFW did not show strong binding activity to the virus in a filter assay, suggesting that DGFW acts through transient binding but leads on to potent inhibitory activity (Figure S2).

Phylogenetic analysis of complete VP1 sequences has identified three EV71 genotypes: A, B and C. Genotypes B and C can be further divided into subgenotypes B1 to B5 and C1 to C5 [18]. DGFW inhibited EV71/BrCr, EV71/TW/2231/98 and EV71/TW/71552/05, which belong to subgenotypes A, C2 and C4, respectively. However, the EC_50_ of DGFW against EV71/TW/1101/08 (subgenotype B5) was greater than 1 mg/mL, but this dose was equivalent to the effective cytotoxic concentration and therefore cannot be considered as having an inhibitory effect. We demonstrated that DGFW targets the virus to inhibit the attachment and entry steps. However, DGFW did not inhibit the B5 subgenotype, and this can be explained by the demonstrated differences in VP1 sequences [19]. The phylogenetic analysis showed distinct differences between CV-B and EV71, and consequently DGFW did not inhibit CV-B1 to B5 as expected (Table 1). Additionally, as DGFW appears to inhibit different genotypes of EV71 to different degrees, this might explain why DGFW does not target cellular factor(s).

Flavonoids are common constituents of plants used in traditional Chinese medicine to treat a variety of diseases. *D. genkwa* has been used for the isolation of flavonoids, and studies have reported the isolation of luteolin, 7-*O*-methylluteolin, tiliroside [20], apigenin, genkwanin, sitosterol, benzoic acid [21] and genkwadaphnin [21,22] from the dried flower buds. Apigenin showed a strong inhibitory activity against xanthine oxidase [21], as well as against severe acute respiratory syndrome coronavirus (SARS-CoV) and influenza virus [23,24,25]. Luteolin and its glycosides have been widely used for their antimicrobial properties, and HBV, SARS-CoV and the influenza virus can be inhibited as shown by *in vitro* studies [24,25,26]. However, no anti-EV71 activity of flavonoids isolated from *D. genkwa* has been demonstrated. Interestingly, similar to our findings, apigenin was able to inhibit the influenza virus but did not show antiviral activity against HSV (Table 1) [23,24,27]. The examination of anti-EV71 activities of apigenin and other purified compounds from *D. genkwa* is ongoing. On a cautionary note, *Daphne* bud extracts, whether from the ether layer or the water layer, can also induce early antigen expression of Epstein–Barr virus (EBV) [28]. Up to 90% of the world’s human population has a latent EBV infection, and it has a high association with nasopharyngeal carcinomas in southeast Asia and certain other countries [29]. In addition, *D. genkwa* possesses the harmful side effect of abortifacient activity. Thus, there is a need to isolate the effective anti-EV71 ingredients that do not lead to the induction of EBV early antigen production or act as abortifacients.

## 3. Experimental Section

### 3.1. Virus and Cell Culture and Viral Amplification

RD cells and human foreskin fibroblast (HFF3) cells were maintained in Dulbecco’s Modified Eagle Medium (DMEM) supplemented with 10% fetal bovine serum (FBS; Gibco BRL, Life Technologies, Grand Island, NY, USA). Madin–Darby canine kidney (MDCK) cells were cultured in DMEM plus 10% FBS, 2 mM l-glutamine (Gibco) and 0.1 mM nonessential amino acids (NEAA; BioWhittaker Inc., Walkersville, MD, USA). Adenocarcinomic human alveolar basal epithelial cells (A549) were cultured in MEM containing NEAA. All cells were kept at 37 °C under 5% CO_2_ in air in a humid incubator. EV71 TW/2231/98 (genotype C), BrCr (genotype A), TW/71552/05 (subgenotype C4), TW/1101/08 (subgenotype B5), coxsackievirus (CV)-B1 to B5, herpes simplex virus (HSV)-1 and adenoviruses were kindly supplied from the Clinical Virology Laboratory of Chang Gung Memorial Hospital, Taiwan (Table 1) [30]. Influenza virus A/WSN/33 was obtained from the American Type Cell Culture and was amplified accordingly [30]. Overnight cultures of monolayers of RD cells were washed with Dulbecco’s phosphate-buffered saline (DPBS) before being inoculated with EV71 or CV-B at a multiplicity of infection (m.o.i.) of 0.01. The cells and culture supernatants containing viral progeny were collected when cells developed a CPE of ~80%. The progeny viruses inside the cells were released by three cycles of freezing–thawing, and the resulting viral suspension was pooled with the culture medium, aliquoted and stored at −80 °C until use.

### 3.2. Plant Material

Dry flower buds of *Daphne genkwa* were supplied and authenticated by the Department of Pharmacy, Chang Gung Memorial Hospital at Chiayi, Taiwan. A voucher specimen (No. CGU-DG-1) was deposited in the herbarium of Chang Gung University, Taoyuan, Taiwan. Dry flower buds (5.0 kg) were extracted with methanol seven times (30 L each) under reflux for 8 h and concentrated to give a brown syrup (745.75 g) (Figure 1). The syrup was suspended in H_2_O and partitioned with CHCl_3_ and *n*-butanol, successively. The water-soluble layer was concentrated to gain a red-brown syrup (153.27 g).

### 3.3. Determination of Median Tissue Culture Infective dose and Antiviral Neutralization Assay

Monolayers of RD cells (2 × 10^4^ per well) were cultured in 96-well plates overnight before use. EV71 TW/2231/98 was used hereafter if not mentioned otherwise. A virus suspension with threefold serial dilution in DMEM containing 2% FBS (E2) was added to the cells and incubated at 37 °C for 72 h and subsequently fixed with 100 μL of 4% paraformaldehyde (PFA) for 2 h. The attached cells were stained with 0.1% crystal violet at room temperature, and the density of staining was measured in a microplate reader (Dynex Technologies, Chantilly, VA, USA). The dilution causing cytopathology in half the cultures (the median tissue culture infective dose, or TCID50) was then calculated using the Reed–Muench method. The TCID50 determinations for the attachment and penetration assays were performed similarly except that preadsorption was carried out for 3 h on ice. A neutralization assay (EC_50_) was used to test the antiviral efficacy of extracts or compounds by measuring the inhibition of CPE induced by enterovirus on RD cells. RD cells (2 × 10^4^ cells/well) were seeded into 96-well plates and incubated for 16–20 h at 37 °C in a 5% CO_2_/air incubator. After infection, cells were overlaid with 50 μL of different concentrations of extracts and incubated at 37 °C in a 5% CO_2_/air incubator for 72 h. At the end of incubation, cells were fixed with PFA, followed by 0.1% crystal violet staining. The concentration required for extracts to reduce the virus-induced CPE by 50% relative to the virus control was defined as the EC_50_.

### 3.4. Cytotoxicity Assay

Aliquots of 2 × 10^4^ RD cells per well were seeded overnight in 96-well plates before use. Twofold serial dilutions of DGFW in E2 were added and incubated at 37 °C for 72 h. The medium was carefully withdrawn without touching the cells. MTT (3-(4,5-dimethylthiazol-2-yl)-2,5- diphenyltetrazolium bromide; 0.5 mg/mL, Sigma-Aldrich, St Louis, MO, USA) was added at 50 μL/well and incubated for 3 h. Dimethylsulfoxide (DMSO; 200 μL/well) was added to dissolve the crystals, and the absorbance at 570 nm of each well was measured using a microtiter plate reader (MRX ELISA reader, Dynex Technologies). The concentration of DGFW that caused the death of 50% of the cells was defined as the 50% cytotoxic concentration (CC_50_).

### 3.5. Plaque Assay

Monolayers of RD cells (4.5 × 10^5^ per well) were seeded in 6-well plates. After an 18 h culture, the cells were inoculated with serially diluted viral suspensions for 1 h. The cells were washed with DPBS and overlaid with warm 0.3% agarose in E2. After incubation for 4 days, the cells were fixed with 4% PFA for 2 h. The agarose was carefully removed and subsequently stained with 0.1% crystal violet. The titer of the virus was expressed as plaque-forming units (PFU) per mL.

### 3.6. Western Blotting

After electrophoresis, proteins were electrotransferred onto polyvinylidene fluoride membranes (Millipore, Billerica, MA, USA). The proteins of interest were incubated with primary antibodies for 1 h and then incubated with appropriate secondary antibodies. Proteins were detected by using an enhanced chemiluminescence western blotting detection system (Millipore). Polyclonal antibodies against 3A and a monoclonal antibody against 3C were as described [31,32].

### 3.7. CPE Testing

RD cells (4 × 10^5^ cells/mL) were seeded into 6-well plates and then challenged with viruses at an m.o.i. of 1. The cells were pretreated with viruses for 1 h on ice and washed with DPBS, and then E2 was added. After 20 h incubation at 37 °C, the cells were stained with 0.4% sulforhodamine B (SRB) in 1% acetic acid, and CPE was observed using a microscope. For DGFW treatment, the cells were pretreated with 0.5 mg/mL extracts and virus simultaneously for 1 h and washed with DPBS, and then E2 containing 0.5 mg/mL DGFW was added. The protective effect exerted by DGFWwas also observed after incubation for 20 h at 37 °C.

### 3.8. Reverse Transcription Polymerase Chain Reaction (PCR) Amplification and Absolute Quantitative (q)PCR

The *in vitro* transcripts of the EV71/2231 infectious clone plasmid, which was kindly provided by Dr. Meishan Ho of Academia Sinica, Taiwan, was serially diluted tenfold for spectrophotometric analysis. After determining the RNA concentration, the copy number of standard RNA molecules can be calculated using the following formula: (X g/μL RNA /[transcript length in nucleotides × 340]) × 6.022 × 1023 = Y molecules/μL. The viral RNA was then mixed with 50 nM forward and reverse primers and SYBR green PCR Master Mix (Applied Biosystems, Foster City, CA, USA) followed by qPCR analysis using an ABI StepOne Plus Sequence Detector System (Applied Biosystems). The primers used were: EV71 VP2 forward, 5´–CTG ATG GCT TCG AAT TGC AA–3´, and reverse, 5´–GCG TTT ATG TAC GGC ACT ATT ATT GT–3´; GAPDH forward, 5´–GGT GGT CTC CTC TGA CTT CAA CA–3´, and reverse, 5´–GCG TCA AAG GTG GAG GAG TG–3´. A standard curve (plot of the CT value/crossing point against the log of the amount of standard added) was thus generated using different dilutions of the standard. RD cells (4 × 10^5^ cells/well) were seeded in 6-well plates overnight. The cells were then preadsorbed with EV71 at an m.o.i. of 5 for 1 h in the presence of 0.5 mg/mL DGFW. The culture medium was then replaced with E2 containing the same concentration of DGFW, and the cells were incubated at 37 °C and harvested at 0, 3, 6 and 9 h post infection (p.i.) for total RNA extraction using TRIzol. cDNA was prepared by reverse transcription with M-MLV RT kits (Invitrogen, Carlsbad, CA, USA) for qPCR analysis using specific viral primers: forward, 5´–CTG ATG GCT TCG AAT TGC AA–3´, and reverse, 5´–GCG TTT ATG TAC GGC ACT ATT ATT GT–3´. Amplification was achieved using the following universal thermal cycling protocol: one cycle at 95 °C for 15 min, 40 cycles at 95 °C for 15 s and 60 °C for 1 min. The absolute amounts of viral RNA were then determined using an external standard curve.

### 3.9. Time-of-Addition Assay

A 6-well tissue culture plate was seeded with RD cells (4 × 10^5^ cells/well), and incubated at 37 °C for 16–20 h under 5% CO_2_ in air. The cells were infected with EV71 at an m.o.i. of 5 on ice. After viral adsorption, the cells were washed with Hank’s buffered salt solution to remove any unbound virus. DGFW (0.5 mg/mL) in E2 was added at −9 to −1 h, −6 to −1 h and −3 to −1 h (preadsorption), −1 to 0 h (adsorption), 0 to 10 h, 3 to 10 h and 6 to 10 h (postadsorption). The supernatants were harvested at 10 h p.i., and the viral titers were determined by plaque-forming assays.

### 3.10. Attachment Assay

A 96-well tissue culture plate was seeded with HFF3 cells (2 × 10^4^ cells/well), which were then incubated overnight at 37 °C under 5% CO_2_ in air. The cells were chilled on ice for 1 h, and the medium was removed. The cells were infected with 100 TCID_50_ of EV71 in the presence of increasing concentrations of DGFW on ice for 3 h. The medium containing the unadsorbed virus was removed, and the cells were then washed three times with DPBS. After incubation for 72 h in E2 at 37 °C, cells were fixed with 4% PFA and stained with crystal violet. Cell viability was determined by measuring the cell density in a microplate reader (MRX ELISA reader, Dynex Technologies). A *Pueraria lobata* extract at 3.125 mg/mL served as a positive control [33]. This attachment method has been described [34].

### 3.11. Penetration Assay

A 96-well tissue culture plate was seeded with HFF3 cells (2 × 10^4^ cells/well), which were then incubated overnight at 37 °C under 5% CO_2_. The cells were chilled on ice for 1 h, and the medium was removed. The cells were infected with 100 TCID_50_ of EV71 on ice for 3 h. The medium containing unbound virus was then removed; various concentrations of DGFW in E2 were added, and the cells were incubated at 37 °C for 1 h to trigger endocytosis of the virus. The infected cells were then treated with alkaline phosphate-buffered saline (PBS; pH 11) for 1 min to inactivate any viruses that had not penetrated the cells, and then acidic PBS (pH 3) was immediately added to neutralize the mix. The neutralized medium was removed, E2 was then replaced, and the cells were incubated at 37 °C for 72 h. The cells were fixed with PFA and subjected to 0.1% crystal violet staining. A *Pueraria lobata* extract at 3.125 mg/mL served as a positive control [33]. This penetration assay was modified from previous reports [35,36].

### 3.12. Data Analysis

Data are expressed as the mean ± standard error of the mean [37] and analyzed using two-tailed Student’s *t*-tests with *p* < 0.05 taken as significant.

## 4. Conclusions

GFW exerts its anti-EV71 effects by inhibiting viral entry without producing cytotoxic side effects and thus provides a potential agent for antiviral chemotherapeutics.

## Supplementary Files

Supplementary File 1: PDF-Document (PDF, 187 KB)

## References

[1] Lin J.Y., Chen T.C., Weng K.F., Chang S.C., Chen L.L., Shih S.R. (2009). Viral and host proteins involved in picornavirus life cycle. J. Biomed. Sci..

[2] Racaniello V.R., Knipe D.M., Howley P.M., Martin M.A., Griffin D.E., Lamb R.A., Roizman B., Straus S.E. (2007). Picornaviridae: The Viruses and Their Replication. Fields Virology.

[3] Chang L.Y., Huang L.M., Gau S.S., Wu Y.Y., Hsia S.H., Fan T.Y., Lin K.L., Huang Y.C., Lu C.Y., Lin T.Y. (2007). Neurodevelopment and cognition in children after enterovirus 71 infection. New Engl. J. Med..

[4] Lin T.Y., Chang L.Y., Hsia S.H., Huang Y.C., Chiu C.H., Hsueh C., Shih S.R., Liu C.C., Wu M.H. (2002). The 1998 enterovirus 71 outbreak in Taiwan: Pathogenesis and management. Clin. Infect. Dis. Off. Publ. Infect. Dis. Soc. Am..

[5] Nishimura Y., Shimojima M., Tano Y., Miyamura T., Wakita T., Shimizu H. (2009). Human P-selectin glycoprotein ligand-1 is a functional receptor for enterovirus 71. Nat. Med..

[6] Yamayoshi S., Yamashita Y., Li J., Hanagata N., Minowa T., Takemura T., Koike S. (2009). Scavenger receptor B2 is a cellular receptor for enterovirus 71. Nat. Med..

[7] Yang B., Chuang H., Yang K.D. (2009). Sialylated glycans as receptor and inhibitor of enterovirus 71 infection to DLD-1 intestinal cells. Virol. J..

[8] Wu B.W., Pan T.L., Leu Y.L., Chang Y.K., Tai P.J., Lin K.H., Horng J.T. (2007). Antiviral effects of Salvia miltiorrhiza (Danshen) against enterovirus 71. Am. J. Chin. Med..

[9] Lin T.Y., Liu Y.C., Jheng J.R., Tsai H.P., Jan J.T., Wong W.R., Horng J.T. (2009). Anti-enterovirus 71 activity screening of chinese herbs with anti-infection and inflammation activities. Am. J. Chin. Med..

[10] Zhou B.N. (1991). Some progress on the chemistry of natural bioactive terpenoids from Chinese medicinal plants. Mem. Inst. Oswaldo Cruz.

[11] Lee M.Y., Park B.Y., Kwon O.K., Yuk J.E., Oh S.R., Kim H.S., Lee H.K., Ahn K.S. (2009). Anti-inflammatory activity of (−)-aptosimon isolated from Daphne genkwa in RAW264.7 cells. Int. Immunopharmacol..

[12] Park B.Y., Min B.S., Ahn K.S., Kwon O.K., Joung H., Bae K.H., Lee H.K., Oh S.R. (2007). Daphnane diterpene esters isolated from flower buds of Daphne genkwa induce apoptosis in human myelocytic HL-60 cells and suppress tumor growth in Lewis lung carcinoma (LLC)-inoculated mouse model. J. Ethnopharmacol..

[13] Hussain K.M., Leong K.L., Ng M.M., Chu J.J. (2011). The essential role of clathrin-mediated endocytosis in the infectious entry of human enterovirus 71. J. Biol. Chem..

[14] Ho H.Y., Cheng M.L., Weng S.F., Chang L., Yeh T.T., Shih S.R., Chiu D.T. (2008). Glucose-6-phosphate dehydrogenase deficiency enhances enterovirus 71 infection. J. Gen. Virol..

[15] McKinlay M.A. (1993). Discovery and development of antipicornaviral agents. Scand. J. Infect. Dis. Suppl..

[16] Phelps D.K., Post C.B. (1995). A novel basis of capsid stabilization by antiviral compounds. J. Mol. Biol..

[17] Shia K.S., Li W.T., Chang C.M., Hsu M.C., Chern J.H., Leong M.K., Tseng S.N., Lee C.C., Lee Y.C., Chen S.J., Peng K.C., Tseng H.Y., Chang Y.L., Tai C.L., Shih S.R. (2002). Design, synthesis, and structure-activity relationship of pyridyl imidazolidinones: a novel class of potent and selective human enterovirus 71 inhibitor. J. Med. Chem..

[18] Brown B.A., Oberste M.S., Alexander J.P., Kennett M.L., Pallansch M.A. (1999). Molecular epidemiology and evolution of enterovirus 71 strains isolated from 1970 to 1998. J. Virol..

[19] Huang S.W., Hsu Y.W., Smith D.J., Kiang D., Tsai H.P., Lin K.H., Wang S.M., Liu C.C., Su I.J., Wang J.R. (2009). Reemergence of enterovirus 71 in 2008 in taiwan: dynamics of genetic and antigenic evolution from 1998 to 2008. J. Clin. Microbiol..

[20] Nikaido T., Ohmoto T., Sankawa U. (1987). Inhibitors of adenosine 3′,5′-cyclic monophosphate phosphodiesterase in Daphne genkwa Sieb. et Zucc. Chem. Pharm. Bull..

[21] Noro T., Oda Y., Miyase T., Ueno A., Fukushima S. (1983). Inhibitors of xanthine oxidase from the flowers and buds of Daphne genkwa. Chem. Pharm. Bull. (Tokyo).

[22] Kasai R., Lee K.H., Huang H.C. (1981). Genkwadaphnin, a potent antileukemic diterpene from Daphne genkwa. Phytochemistry.

[23] Wu Q., Yu C., Yan Y., Chen J., Zhang C., Wen X. (2010). Antiviral flavonoids from Mosla scabra. Fitoterapia.

[24] Liu A.L., Liu B., Qin H.L., Lee S.M., Wang Y.T., Du G.H. (2008). Anti-influenza virus activities of flavonoids from the medicinal plant Elsholtzia rugulosa. Planta Med..

[25] Ryu Y.B., Jeong H.J., Kim J.H., Kim Y.M., Park J.Y., Kim D., Nguyen T.T., Park S.J., Chang J.S., Park K.H., Rho M.C., Lee W.S. (2010). Biflavonoids from Torreya nucifera displaying SARS-CoV 3CL(pro) inhibition. Bioorg. Med. Chem..

[26] Tian Y., Sun L.M., Liu X.Q., Li B., Wang Q., Dong J.X. (2010). Anti-HBV active flavone glucosides from Euphorbia humifusa Willd. Fitoterapia.

[27] Ozcelik B., Kartal M., Orhan I. (2011). Cytotoxicity, antiviral and antimicrobial activities of alkaloids, flavonoids, and phenolic acids. Pharm. Biol..

[28] Zeng Y., Zhong J.M., Ye S.Q., Ni Z.Y., Miao X.Q., Mo Y.K., Li Z.L. (1994). Screening of Epstein-Barr virus early antigen expression inducers from Chinese medicinal herbs and plants. Biomed. Environ. Sci..

[29] Niedobitek G. (2000). Epstein-Barr virus infection in the pathogenesis of nasopharyngeal carcinoma. Mol. Pathol..

[30] Hsu J.T., Yeh J.Y., Lin T.J., Li M.L., Wu M.S., Hsieh C.F., Chou Y.C., Tang W.F., Lau K.S., Hung H.C., Fang M.Y., Ko S., Hsieh H.P., Horng J.T. (2012). Identification of BPR3P0128 as an Inhibitor of Cap-Snatching Activities of Influenza Virus. Antimicrob. Agents Chemother..

[31] Tang W.F., Yang S.Y., Wu B.W., Jheng J.R., Chen Y.L., Shih C.H., Lin K.H., Lai H.C., Tang P., Horng J.T. (2007). Reticulon 3 binds the 2C protein of enterovirus 71 and is required for viral replication. J. Biol. Chem..

[32] Weng K.F., Li M.L., Hung C.T., Shih S.R. (2009). Enterovirus 71 3C protease cleaves a novel target CstF-64 and inhibits cellular polyadenylation. PLoS Pathog..

[33] Su F.M., Chang J.S., Wang K.C., Tsai J.J., Chiang L.C. (2008). A water extract of Pueraria lobata inhibited cytotoxicity of enterovirus 71 in a human foreskin fibroblast cell line. Kaohsiung J. Med. Sci..

[34] de Logu A., Loy G., Pellerano M.L., Bonsignore L., Schivo M.L. (2000). Inactivation of HSV-1 and HSV-2 and prevention of cell-to-cell virus spread by Santolina insularis essential oil. Antivir. Res..

[35] Albin R., Chase R., Risano C., Lieberman M., Ferrari E., Skelton A., Buontempo P., Cox S., DeMartino J., Wright-Minogue J., Jirau-Lucca G., Kelly J., Afonso A., Kwong A.D., Rozhon E.J., O’Connell J.F. (1997). SCH 43478 and analogs: in vitro activity and *in vivo* efficacy of novel agents for herpesvirus type 2. Antivir. Res..

[36] Rosenthal K.S., Perez R., Hodnichak C. (1985). Inhibition of herpes simplex virus type 1 penetration by cytochalasins B and D. J. Gen. Virol..

[37] Bedard K.M., Semler B.L. (2004). Regulation of picornavirus gene expression. Microbes Infect. Inst. Pasteur.

