# Supplementary material for: Daphne Genkwa Sieb. et Zucc. Water-Soluble Extracts Act on Enterovirus 71 by Inhibiting Viral Entry
Source: Viruses. 2012 Apr 11;4(4):539–56. doi: 10.3390/v4040539 (PMC3347322; doi:10.3390/v4040539)

Figure S1. Titration of cytotoxicity of DGFW on RD cells.

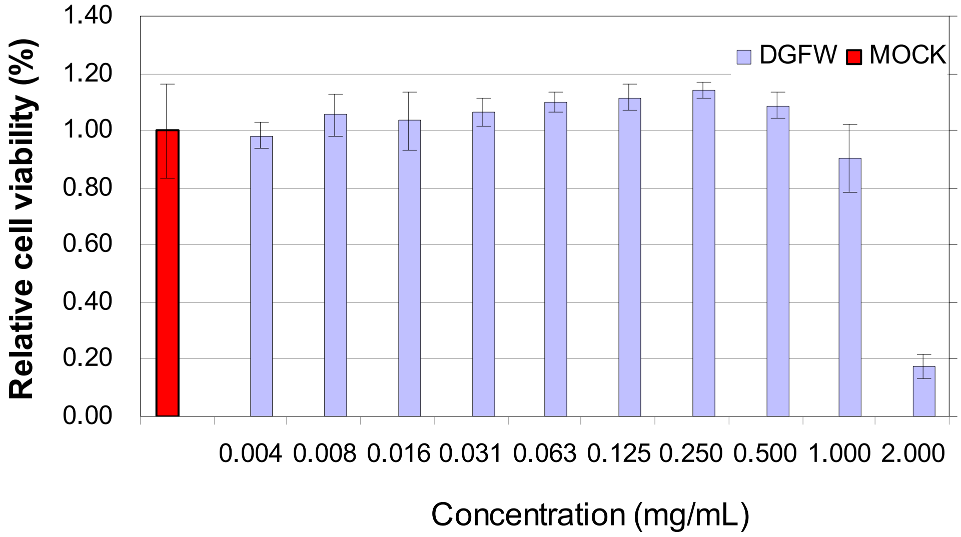

Figure S2. DGFW did not bind to virus directly, as shown by a filter assay using an Amicon centrifugal device. DGFW (2 mg/mL) in a total volume was placed in the reservoir of an Amicon Ultra-15 centrifugal filter unit (Ultra 100K; Millipore) and centrifuged at 3500 *g* for 5 min. The resulting concentrated solution was diluted with DMEM up to 2.5 mL and centrifuged again. The anti-EV71 activities of the retentate and filtrate were measured using a neutralization (inhibition of CPE) assay (A–C). The majority of the bioactive ingredients were in the first round filtrate and none in the second round filtrate (A and B) or retentate (C). (D) The virus stock (1×10^8 PFU/mL) was mixed with DGFW (2 mg/mL) or DMSO to a total volume of 2.5 mL and centrifuged twice as described. The remaining viral titers were measured. The virus treated with DGFW did not show any reduction of viral titer. This is one representative result from two experiments.

(A) Filtrate layer 1

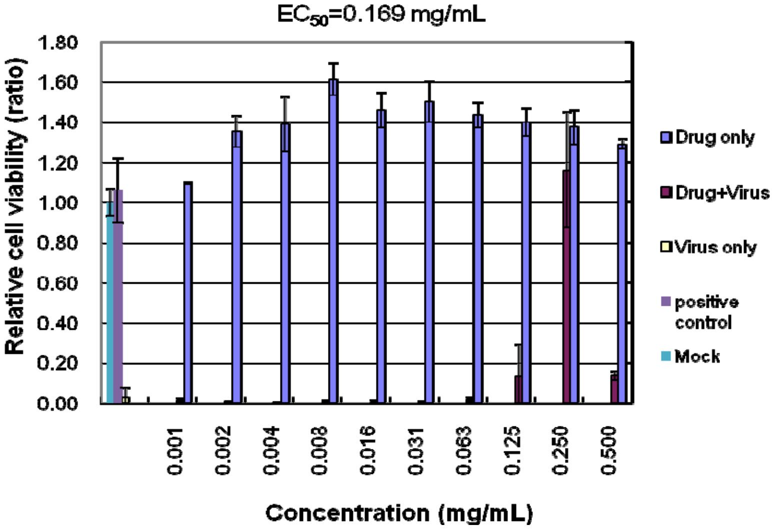

(B) Filtrate layer 2

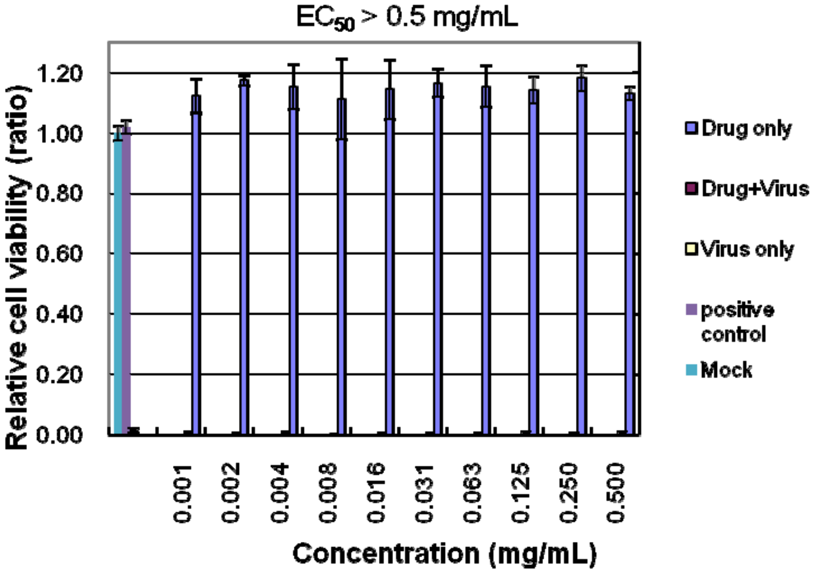

(C) Final retentate layer

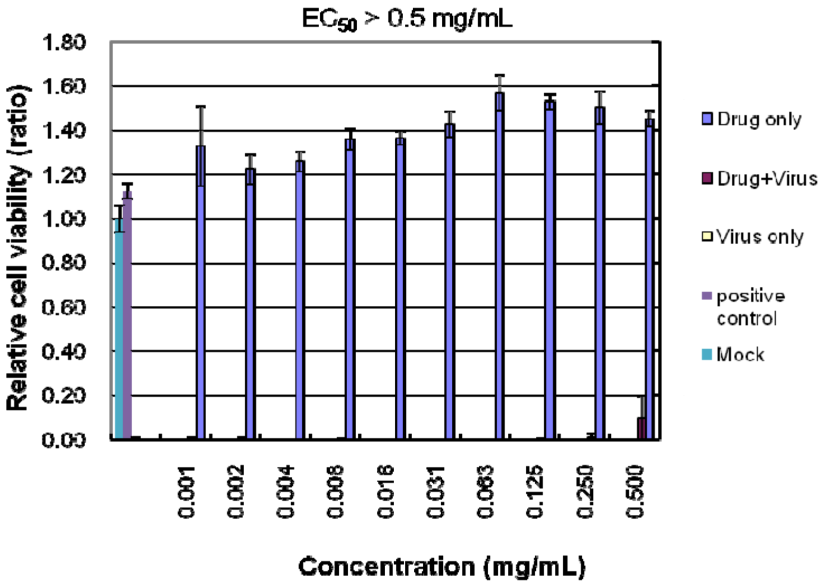

(D) Remaining viral titer in the retentate

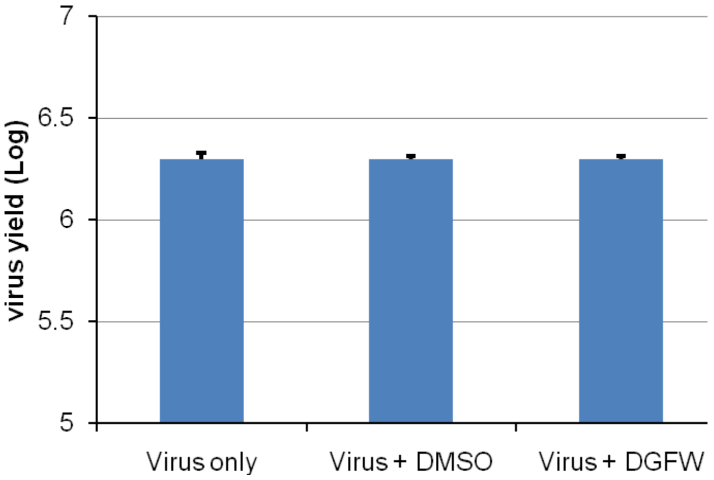

Supplement: Supplementary File 1: — PDF-Document (PDF, 187 KB) [file viruses-04-00539-s001.pdf]
